# Development and Characterization of Clindamycin-Loaded Dextran Hydrogel for Controlled Drug Release and Pathogen Inhibition

**DOI:** 10.3390/gels12010082

**Published:** 2026-01-17

**Authors:** Iqra Jawad, Asma Rehman, Mariam Hamdan, Kalsoom Akhtar, Shazia Khaliq, Munir Ahmad Anwar, Nayla Munawar

**Affiliations:** 1 Industrial Biotechnology Division, National Institute for Biotechnology and Genetic Engineering College, Pakistan Institute of Engineering and Applied Sciences (NIBGE-C, PIEAS), Faisalabad 38000, Pakistan; iqrajawad_786@yahoo.com (I.J.); asmanano@gmail.com (A.R.); kalsoom1967@gmail.com (K.A.); skhaliq1976@gmail.com (S.K.); 2 Department of Chemistry, College of Science, United Arab Emirates University (UAEU), Al Ain 15551, United Arab Emirates; 202050038@uaeu.ac.ae

**Keywords:** hydrogel, Exopolysaccharides, dextran, drug delivery, clindamycin

## Abstract

The naturally occurring, biocompatible and biodegradable biopolymer dextran is a versatile material for the formulation of hydrogels with desirable properties for use in medicine, drug delivery, and tissue engineering applications. The distinctive structural and physicochemical characteristics, such as polymeric nature, gelling ability and excellent swelling properties, present it as an excellent biomaterial for drug delivery. This study explores the synthesis and characterization of dextran hydrogel for the encapsulation of clindamycin as an innovative approach for controlled drug delivery. The dextran hydrogel was synthesized through a simple and cost-effective method, and its swelling behavior, temperature and pH dependence, and surface morphology were investigated. The maximum equilibrium swelling ratio (73 ± 1%) of the hydrogel was observed in water at 25 °C within 120 min, and the hydrogel was found to be pH- and temperature-dependent for more precise and targeted drug delivery. Moreover, the dextran hydrogel was found to retain water for up to 18 h and remain stable for 8 days. The presence of a roughened surface with large openings/pores on the surface illustrated the high swelling capability of the synthesized hydrogel. In addition, the dextran hydrogel loaded with clindamycin demonstrated high drug loading capacity (70 ± 2%), rapid (65 ± 2%) in vitro drug release potential and pathogen-inhibitory activity against *Staphylococcus gallinarium* and *Bacillus subtilis*.

## 1. Introduction

Biotechnological and pharmaceutical companies are increasingly focusing on targeted drug delivery systems to improve drug stability and half-life while reducing dosing frequency. The ideal drug carriers must be biocompatible, biodegradable, and capable of protecting the drug from degradation in the body [1]. Exopolysaccharides (EPSs) are highly promising materials for intelligent therapeutics due to their Generally Regarded As Safe (GRAS) status and diverse structural and functional features [2]. Moreover, the highly hydrophilic, biodegradable, rheological and biocompatible characteristics of microbial EPSs present them as highly suitable carriers for biomedical applications. The release rate of encapsulated drugs can be influenced by variation in pH, temperature, bio-adhesion, or biodegradability of the carrier matrix [3].

EPS-based drug carrier systems exhibit multiple advantages over conventional drug delivery approaches. The traditional approach often relies on the creation of pH-sensitive coatings which are highly sensitive to lumen pH, meal composition, gastric emptying, and individual differences in GI fluid conditions. High-fat meals and variable gastric acidity often cause premature or delayed drug release in such pH-dependent systems [4]. In contrast, EPS-based carriers rely primarily on microbiota-mediated degradation in the colon rather than environmental pH, making them substantially less affected by food intake or GI variability than conventional pH-triggered systems [5,6]. Their physicochemical stability across a wide pH range (2–8), high resistance to digestive enzymes, and minimal interaction with luminal components make them less susceptible to dietary fluctuations [7,8]. EPS-based encapsulation enhances targeted delivery and drug stability until the colon, reducing the required therapeutic dose and minimizing premature release in the upper GIT [7]. EPSs can be readily altered chemically and biochemically due to the presence of several reactive groups in their structure. Their reactive functional groups allow easy chemical or enzymatic modification, while hydrophilic groups (hydroxyl, carboxyl, amino) improve bio-adhesion to epithelial and mucosal surfaces [2,9]. EPS polymers also offer improved pharmacokinetics, excellent safety, low toxicity, and lower production costs compared to many other polymers that require labor-intensive downstream processing [10,11].

The skin acts as a protective barrier and maintains fluid, body hydration and electrolyte balance. Wounds compromise this barrier and are highly susceptible to bacterial and fungal infections, which can delay healing and, if untreated, lead to chronic, hard-to-heal wounds [2]. This could be due to the excessive Reactive Oxygen Species (ROS) generated in the onset of bacterial infection, sustaining inflammation and significantly delaying wound healing [12,13,14]. Despite the fact that there are various ways to prevent and treat wound infection clinically, the local application of antimicrobials or antibiotics is still a successful strategy [15,16]. Biological and biosynthetic wound dressings are widely accepted for their safety and unique properties, with hydrogels being particularly effective and preferred over alternative dressings [14,17]. This three-dimensional polymeric network absorbs large amounts of water or biological fluids, enhancing drug or antibiotic bioavailability while reducing cytotoxicity [18,19]. Due to the polymeric structure of EPSs, they can be easily formulated as hydrogels by using crosslinking or other physical procedures and are the best candidates for tissue engineering and controlled release of drugs [20,21].

Among EPSs, dextran is an attractive biocompatible biomaterial with unique physicochemical properties and functional versatility, making it a promising carrier for developing next-generation therapeutic delivery systems. It is primarily composed of linear 1,6-linked D-glucopyranose residues [22,23], possesses strong water binding capacity, inhibits platelet over-activation, and serves as a moderate reactive oxygen radical scavenger [14,24]. Dextran hydrogels promote angiogenesis, provide antimicrobial activity, support skin regeneration, reduce inflammation [25], enhance drug stability, limit systematic accumulation in the blood and enable growth factor delivery [10].

The present study aims to develop a dextran hydrogel as an advanced alternative to conventional clindamycin delivery platforms, offering controlled release, enhanced stability, and targeted antimicrobial action. Traditionally, clindamycin is administered through oral and intravenous (IV) delivery as a standard for treating diverse bacterial infections. While oral dosing ensures systemic drug exposure and IV administration provides rapid therapeutic levels in moderate-to-severe cases, these approaches are frequently associated with systemic toxicity and necessitate repeated dosing. Topical clindamycin formulations (gels, lotions, foams) are also widely used for localized dermatological infections, but their poor retention, rapid dilution by wound exudates, and short residence time at the application site limit their clinical efficacy [26,27]. These constraints highlight the need for more sustained drug-delivery systems. Dextran hydrogels overcome these limitations by offering excellent biocompatibility, moisture retention, prolonged drug release, and strong adhesion to moist wound surfaces. Their biodegradable and ECM-mimetic properties further promote wound healing and tissue regeneration [28,29].

In this work, dextran-based hydrogels were formulated and assessed for their suitability as carriers for clindamycin, selected as a model therapeutic agent. Key parameters, including drug-loading efficiency, in vitro release kinetics, and functional performance, were systematically evaluated. Furthermore, the antimicrobial efficacy of clindamycin-loaded dextran hydrogels was assessed against *S. gallinarum* and *B. subtilis* to determine their pathogen inhibition potential. To the best of our knowledge, this is the first study to report clindamycin loading into a dextran hydrogel using a simple, cost-effective, and reproducible crosslinking strategy based on native microbial dextran. Unlike previously reported systems that rely on complex chemical modifications, multiple polymer blends or expensive crosslinking strategies, the present system utilizes native microbial dextran and a straightforward alkaline crosslinking route. This platform integrates hydrogel fabrication, physicochemical characterization, drug loading and release, degradation behavior, and antibacterial efficacy within a single system. The developed hydrogel demonstrates high drug-loading efficiency, rapid initial release suitable for infected wounds, and effective inhibition of Gram-positive pathogens. Collectively, this work highlights the potential of the developed hydrogel as a scalable and clinically relevant platform for topical and localized infection management, wound healing, and controlled drug delivery, particularly in resource-limited settings.

## 2. Results and Discussion

### 2.1. Synthesis of Dextran Hydrogel

**Figure 1:**
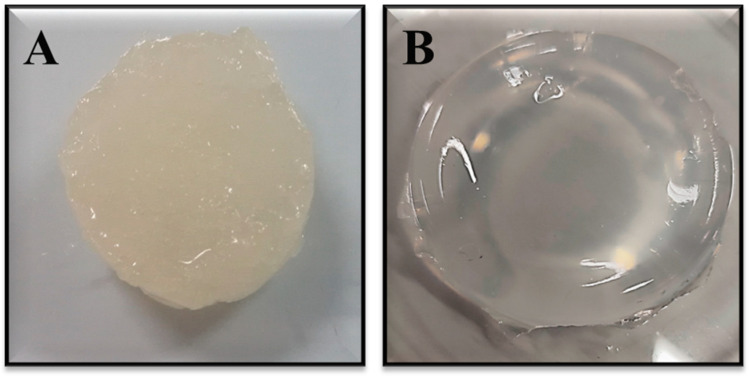
Formulation of dextran hydrogel using MBA crosslinker. (**A**) Dextran hydrogel after synthesis and before washing; (**B**) dextran hydrogel after washing.

The dextran hydrogel was prepared by a polymerization reaction between completely dissolved dextran in an alkaline solution and a crosslinker *N*,*N*′-methylenebisacrylamide (MBA). After 10 min following continuous stirring, the solution started to convert into gel form. It was observed that complete gelation occurred after the incubation of dextran hydrogel for 3 h at 25 °C. A temperature above 30 °C was found to restrict the gelation process and decrease hydrogel formation. The visual observation of dextran hydrogel revealed that the initially formed hydrogel after the polymerization of dextran appeared milky and viscous due to the presence of unreacted NaOH and other substances. However, after being washed with deionized water for three days, it was transformed into a transparent, stable, and homogenized hydrogel due to the leaching of unreacted compounds (Figure 1). The final pH of the prepared hydrogels was measured after the neutralization and washing steps using a calibrated pH meter. The pH was found to be in the range of 6.8–7.2, confirming complete neutralization of the NaOH.

Diverse EPSs, including dextran, chitosan, alginate, cellulose, etc., are used as such or subjected to required modifications for the formation of hydrogels (the chemical structure of dextran is provided in the supplementary data as Figure S1). The synthesis process has a big impact on the characteristics of hydrogels. Dextran hydrogel has previously been prepared utilizing a number of physical and chemical techniques, including freezing and thawing, covalent crosslinking, and radiation [17]. In another study, dextran was formulated as a hydrogel by creating a polymeric network using MBA crosslinker [30]. This work followed a similar methodology with some modifications to prepared the dextran hydrogel by polymerization reaction. The reason behind this formulation is that the hydroxyl part of the dextran was activated under alkaline conditions and was polymerized by the crosslinking agent through covalent interactions, resulting in the formation of a three-dimensional dextran hydrogel network [31].

### 2.2. Optimization of Crosslinker Concentrations for the Synthesis of Dextran Hydrogel

**Figure 2:**
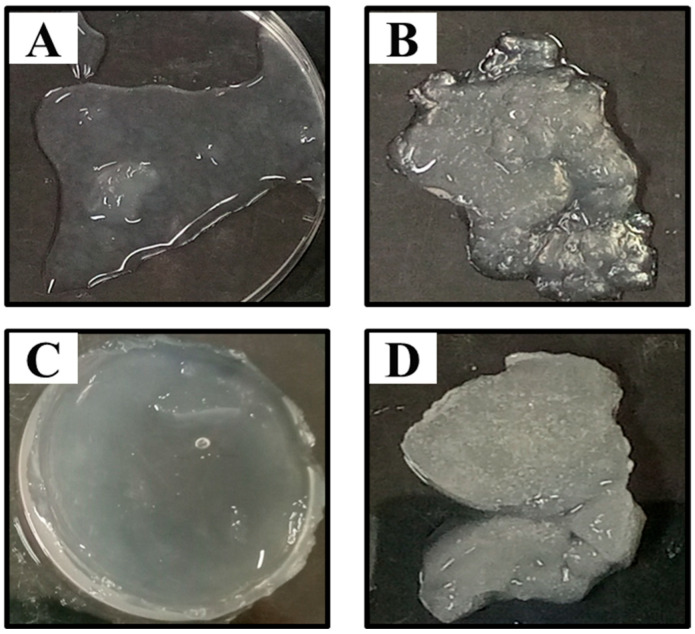
Physical appearance of dextran hydrogels synthesized using different concentrations of MBA swollen in water. (**A**): 0.20:1 (DexH1); (**B**): 0.35:1 (DexH2), (**C**): 0.5:1 (DexH3), and (**D**): 0.75:1 (DexH4).

In order to optimize the concentration of crosslinker (MBA) for the synthesis of dextran hydrogels, four different MBA/dextran ratios including 0.20:1 (DexH1), 0.35:1 (DexH2), 0.5:1 (DexH3), and 0.75:1 (DexH4) were tested. The visual observation revealed that DexH3 was mostly homogenized hydrogel, having a crosslinker ratio of 0.5:1 (Figure 2). The degree of polymerization was decreased in DexH2 hydrogel, whereas dextran was not polymerized into gel form at the crosslinker ratio of 0.20 in DexH1. At a ratio of more than 0.5, polymerization did occur with decreased homogeneity.

### 2.3. Water Absorbency and Swelling Behavior of Dextran Hydrogel

**Figure 3:**
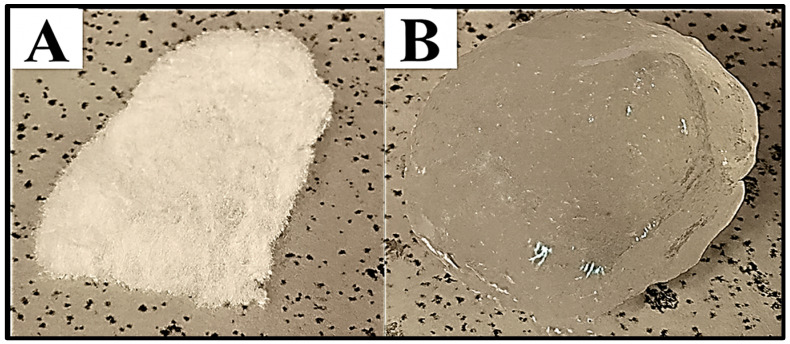
Analysis of physical appearance of dextran hydrogels (**A**): dried (**B**): swollen in water.

**Figure 4:**
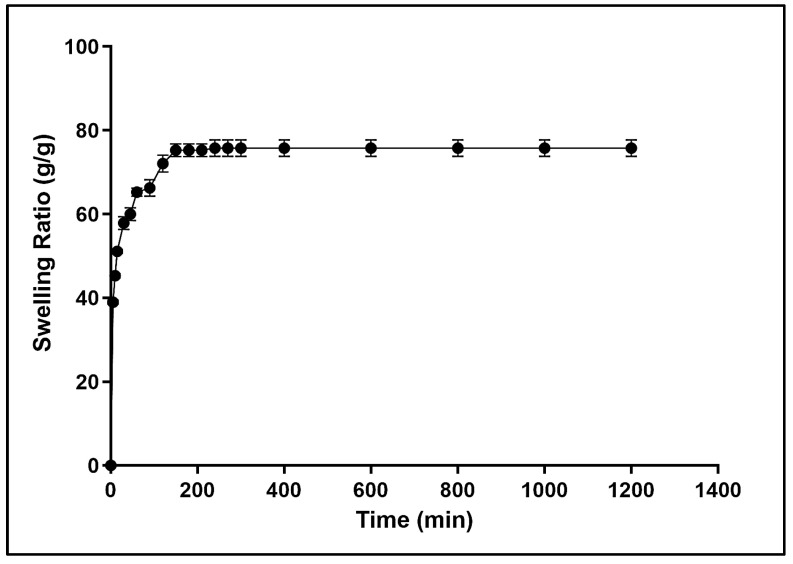
Analysis of the swelling behavior of dextran hydrogels in dIH_2_O. All experiments were performed in triplicate (*n* = 3). The data is presented as mean ± SEM.

The hydrogel’s applicability as a sorbent material or a biomaterial depends on its rate and water absorption capacity. Due to the hydrophilic nature of dextran, the swelling behavior of the dextran hydrogel was assessed in deionized water at 30 °C. The results revealed that the dextran hydrogel possessed strong water absorption ability. The dried piece of hydrogel was observed as a transparent and stable hydrogel after swelling (Figure 3). Following immersion in deionized water, a rapid increase in the swelling ratio occurred within 5 min, whereas almost 50% swelling was achieved within 15 min. The hydrogel was observed to reach its highest equilibrium degree of swelling (73 ± 1) within 120 min of immersion, followed by the plateauing of the swelling ratio up to 180 min (Figure 4). The swelling behavior was observed for 10 h, but no change in the equilibrium swelling ratio was observed.

It is crucial to look into how well hydrogels swell because of their distinctive three-dimensional network structure, which allows them to absorb a lot of water while remaining undissolved [32]. The most crucial aspects of hydrogels used as drug delivery systems are their water absorption and water retention capacities and thus, the performance of the produced dextran hydrogels in terms of swelling and deswelling was comprehensively studied. In the present study, initially, deionized water with a neutral pH was used to measure the swelling qualities in order to reduce the influence of ions. This property could be attributed to the crosslinking density of gels as well as the hydrophobic and hydrophilic characteristics of the used polymer. The higher swelling capacity of dextran hydrogel represented the lower crosslinking density of the gel and it is considerably higher than previously reported data, in which polydopamine-incorporated dextran hydrogels exhibited a maximum degree of swelling between 25 and 30 [31]. The swelling capacity of the unmodified dextran hydrogels crosslinked with MBA was significantly higher in comparison to the reported range of highest swelling ratio of another EPS, levan hydrogels, which was found to be 7.1 ± 0.3 for unmodified levan hydrogels crosslinked with BDDE [17] and 5.5 for the temperature-responsive levan/nisopropyl acrylamide (levan/pNIPA) hydrogels [33]. The swelling percentage of hydrogels is associated with the counterion osmotic pressure, which is influenced by the dissociation of the ionic groups in the aqueous solution. It is also known that the gel counterion interacts electrostatically with its spatial neighbors in addition to within itself, which affects the gel’s ability to swell. As a result, hydrogels’ percentage of swelling depends on the level of dissociation and the concentration of the gel electrolytes [32].

### 2.4. Effect of Buffer on the Swelling of Dextran Hydrogel

**Figure 5:**
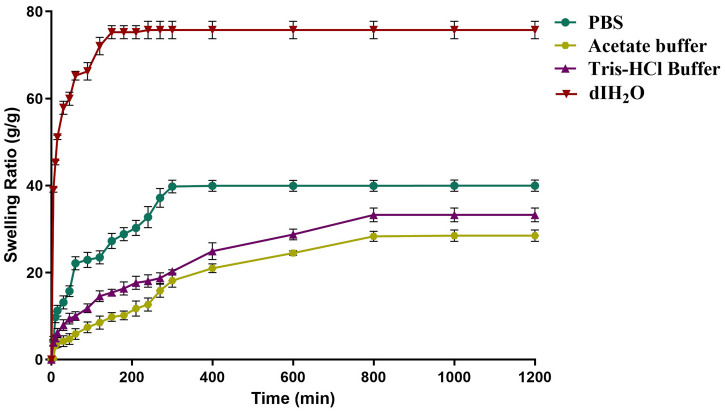
Swelling behavior of dextran hydrogels in different buffer solutions in comparison with dIH_2_O. All experiments were performed in triplicate (*n* = 3). The data is presented as mean ± SEM.

The swelling behavior of dextran hydrogels was also investigated in different surrounding fluids with a different pH in comparison with deionized water. The results revealed that the equilibrium swelling degree and the rate of swelling are pH-dependent. The highest degree of swelling (73.0 ± 1) was observed in deionized water within 120 min of immersion, with the 50% swelling of dextran hydrogel in 10 min. The results revealed that the pH of the buffer caused a pronounced effect on the swelling behavior of the dextran hydrogel. Among three tested buffers, the maximum swelling ratio of 38.0 ± 2 was achieved after 5 h of immersion in PBS buffer with pH 7.4, which is almost half that of deionized water. Any decrease or increase in this pH led to a decrease in the water absorbing capacity of the hydrogel and increased the time to attain the equilibrium swelling ratio. Comparatively, this time was increased for the dextran hydrogels that were swollen in acetate and Tris HCl buffers and equilibrium swelling ratios of 27 ± 2 and 31 ± 2 were attained in 800 min (Figure 5). These results indicated that the dextran hydrogel exhibited the highest degree of swelling in neutral pH, which makes it a useful biocompatible material for colon-targeted drug delivery.

The swelling behavior of dextran hydrogels is influenced by several factors, including the degree of crosslinking, polymer concentration, molecular weight of dextran, surrounding fluid and environmental conditions such as temperature. Modifications to these conditions can help fine-tune the swelling properties to meet specific requirements for different applications. In the present study, the swelling kinetics of the hydrogels were found to be pH-dependent as well. The maximum swelling ratio was achieved in deionized water due to the hydrophilic nature of dextran, whereas PBS at neutral pH exhibited half of the swelling compared to dIH_2_O. This phenomenon could be explained due to the interference of ions or the composition of the PBS buffer. The presence of salts in PBS increases the ionic strength, which may decrease the swelling capacity of the hydrogels due to increased electrostatic interactions [34]. These salts can create an osmotic pressure that opposes the hydrogel’s swelling. In contrast, water has a lower osmotic pressure, which allows the hydrogel to absorb more water and swell to a larger extent. At pH 7, water molecules undergo hydrogen bonding and so generate more space for the molecules to penetrate inside and swell more. Dextran is a polysaccharide with hydroxyl groups, some of which can ionize in water [35]. The presence of ions in PBS might alter the charge interactions between the hydrogel and the surrounding solvent, affecting the swelling behavior.

In addition to these findings, it was shown that dextran hydrogel takes a longer time to reach the equilibrium swelling degree and swells less in acidic or basic pH conditions. A possible reason could be the ionization of dextran molecules and the interactions between the hydrogel network and surrounding solvent molecules. Specifically, the hydroxyl group of dextran can undergo protonation at low pH, forming –OH_2_^+^ groups, and deprotonation at high pH, forming –O^−^ groups. This ionization leads to electrostatic attraction or repulsion with solvent molecules, which reduces the swelling of the hydrogel. Another possible explanation could be that the ionized –OH groups may create weaker hydrogen bonds under acidic or basic circumstances, which would lower water uptake and, in turn, reduce the hydrogel’s swelling [35,36,37].

### 2.5. Effect of Temperature on the Swelling of the Dextran Hydrogel

**Figure 6:**
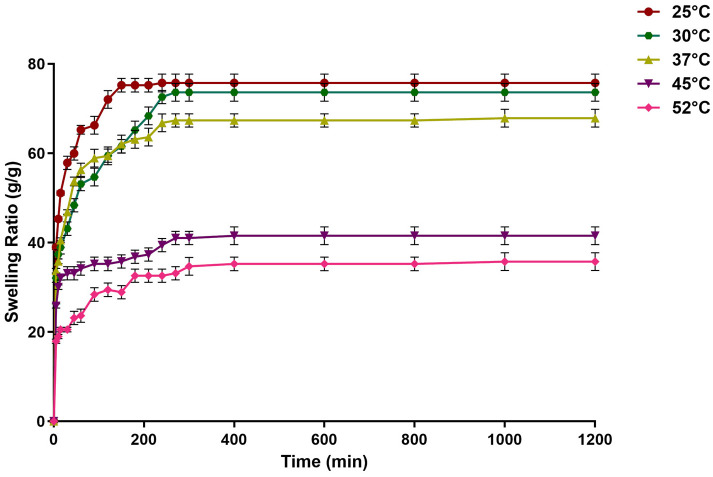
Swelling behavior of dextran hydrogels at different temperatures in dIH_2_O. All experiments were performed in triplicate (*n* = 3). The data is presented as mean ± SEM.

The temperature dependence of the dextran hydrogel was assessed by investigating its swelling behavior at different temperature ranges from 25 °C to 52 °C. The results showed the temperature dependence of dextran hydrogel swelling. It was found that the dextran hydrogels exhibited a maximum equilibrium swelling ratio of 75 ± 1 after 120 min of swelling at 25 °C, as shown in Figure 6. A slight decline in the swelling behavior of the dextran hydrogel was observed at 30 °C and 37 °C. Increasing the temperature beyond 37 °C led to an abrupt decrease in the swelling ratio, exhibiting the lowest swelling ratio (35 ± 1) at 55 °C (Figure 6).

It is reported that dextran hydrogel swells due to the presence of hydrophilic functional groups, which have an affinity for water molecules. However, at higher temperatures, water molecules also gain kinetic energy, leading to weaker interactions with the hydrogel network. As a result, the dextran chains may not retain water as effectively, leading to reduced swelling. When the temperature rises, temperature-sensitive hydrogels can either show a positive temperature response, in which case the gel material swells, or a negative temperature reaction, in which case the gel material shrinks. In the present investigation, the rise in temperature led to reduced swelling. A possible explanation could be that the increase in temperature cause a reversal in the difference in osmotic pressure and water escapes the hydrogel, causing it to shrink. On the other hand, a drop in temperature causes a rise in the osmotic pressure difference, which allows water to enter the hydrogel and causes swelling [38].

### 2.6. Deswelling and Water Retention Capacity of Dextran Hydrogel

**Figure 7:**
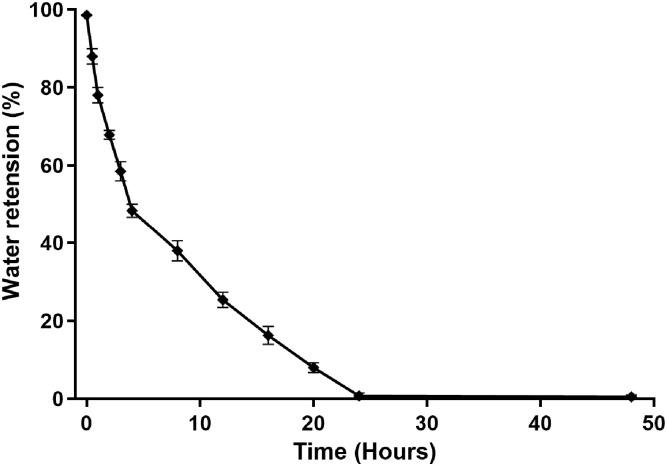
Water retention capacity of dextran hydrogel at 37 °C. All experiments were performed in triplicate (*n* = 3). The data is presented as mean ± SEM.

The deswelling and water retention capacity of the dextran hydrogel was tested by dehydrating the gel at 37 °C. The results showed that a rapid decrease (15%) in water retention occurred within the initial 30 min of dehydration. The deswelling rate continued to increase and 50% water retention was observed after 3 h. After that, the dehydration rate become slightly lower and the gel was completely dehydrated after 18 h of incubation at 37 °C (Figure 7).

Along with the ability to swell, hydrogels also displayed the ability to retain water or moisture within its crosslinked structure. The degree of crosslinking, according to the earlier literature, is a component that affects how much water a hydrogel can hold [32]. The results showed that the hydrogel was fully dehydrated after 18 h. It can be commented that the synthesized hydrogel exhibited an extent of crosslinking which allowed it to hold water for a long time. Similar to a previous study which described the stability of TOB/Dex-CHO/SD in PBS at pH 7.4 [21], the dextran hydrogel formulated in the present study was also found to be stable at pH 7.4 for 7 days.

### 2.7. Stability Analysis of Dextran Hydrogel

**Figure 8:**
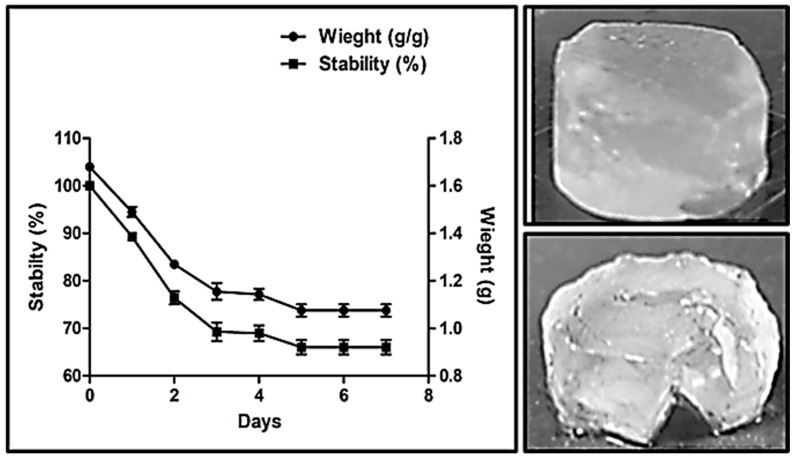
Stability analysis of dextran hydrogel recorded at 37 °C in PBS. All experiments were performed in triplicate (*n* = 3). The data is presented as mean ± SEM.

The stability of dextran hydrogel was tested in 20 mL of PBS at 37 °C by measuring the weight of the hydrogel every 24 h. The PBS was changed every 24 h. The results show that dextran hydrogel showed only 10% degradation after 24 h, followed by up to 30% degradation after 4 days. An equilibrium in degradation was observed on day 5 of the experiment, at which the hydrogel was degraded by 35%. The degradation of the hydrogel was observed for 7 days, after which no further decline in the weight of the hydrogel was observed (Figure 8).

This degradation profile demonstrates that the hydrogel maintains its structural integrity during the critical early stages, which is important for wound protection and initial therapeutic delivery. The gradual degradation observed thereafter provides sustained release potential, allowing the hydrogel to deliver active agents over several days while maintaining a moist environment conducive to healing. The equilibrium observed after day 5 indicates that the hydrogel does not undergo uncontrolled breakdown, minimizing the risk of frequent dressing changes that could disrupt tissue regeneration. Overall, the results highlight that the dextran hydrogel combines stability with controlled degradability—key attributes for an effective wound dressing that balances durability with sustained therapeutic function.

### 2.8. Scanning Electron Microscopy (SEM) Analysis

**Figure 9:**
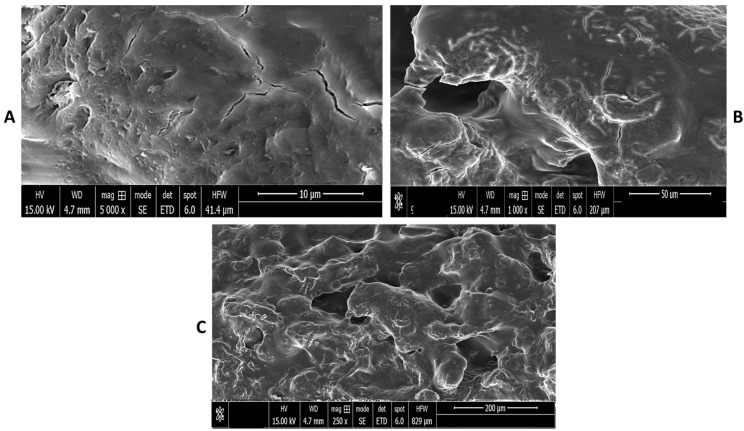
Images of SEM analysis of dextran hydrogel. SEM images showing the surface morphology of dextran–MBA hydrogel at different magnifications (**A**): 5000×, (**B**): 1000× and (**C**): 250×. Samples were sputter-coated with a 5–10 nm gold layer prior to imaging. All micrographs represent major surface views. Scale bars: (**A**): 10 μm, (**B**): 50 μm, and (**C**): 200 μm. For sample preparation, the freeze-dried hydrogels were coated with a thin film (5–10 nm) of gold.

Scanning electron microscopy was used to analyze the morphology of the dextran hydrogels. The outcomes demonstrated the large surface area of the dextran hydrogel. The surface of the hydrogel was clearly observed as rough with the presence of some cracks and openings (Figure 9). These pores may be due to the presence of ionic and hydrophilic constituents in dextran. Further, these openings confer high swelling and deswelling properties that subsequently enhance drug encapsulation and release under specific conditions. Deeper analysis of the SEM image indicated the presence of surface openings or voids which are vital to increase the drug loading capacity and facilitate the faster swelling of dextran hydrogel. As previously reported, the hydrogels’ porous nature lends itself to their usefulness as wound dressing materials due to their water and oxygen permeability as well as their capacity for diffusion [17]. These outcomes supported the potential use of dextran hydrogels in biomedical applications.

### 2.9. Drug Loading Studies

#### 2.9.1. Estimation of Clindamycin Loading Capacity in Dextran Hydrogel

**Figure 10:**
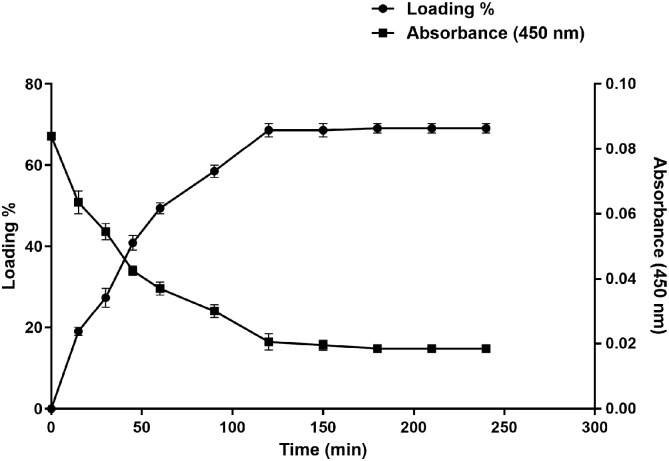
Analysis of clindamycin loading capacity of dextran hydrogels in dIH_2_O. All experiments were performed in triplicate (*n* = 3). The data is presented as mean ± SEM.

The drug loading capacity of dextran hydrogels was evaluated using a clindamycin antibiotic as a pharmaceutically active substance. For this purpose, a dried piece of dextran hydrogel was placed in 100 mL of the 3 mg/mL antibiotic solution. The weight of the hydrogel was tested at different time points to investigate the swelling of the hydrogel in a drug solution, whereas the drug loading capacity was estimated by comparing the absorbance values with the standard curve of clindamycin. Rapid drug loading was observed within 60 min of soaking, which resulted in the loading of about 50% of the drug. The dextran hydrogel showed about 70 ± 2% drug loading capacity after soaking for 2 h in a clindamycin solution. The drug loading capacity continued to be assessed for four hours, but no more drug absorption was observed after 120 min (Figure 10).

The combination of polymers and drugs in hydrogels can be formulated as single-drug-loaded or double-drug-loaded gels, where the combination of two drugs can provide enhanced bactericidal effects [21]. For instance, levan-based hydrogels were developed for the encapsulation and release of 5- aminosalycylic acid (5-ASA), a bioactive compound effective for ulceritis. Moreover, biocompatible levan was also created in the form of a hydrogel as a dermal filler for soft tissue supplements. Dextran hydrogels have also been claimed to be utilized for the controlled release of proteins [39]. Various dextran-based formulations have been devised for the development of efficient drug delivery systems. For instance, dextran-based fibers were obtained by photo-crosslinking methacrylated dextran with gelatin, and are reportedly potential candidates for soft tissue engineering [40,41]. Previous investigations have reported that dextran molecules carry a large number of hydroxyl groups for the incorporation of drugs, while the release of drugs from dextran-based fibers varies depending upon the molecular weight of dextran [42].

Clindamycin is a strong broad-spectrum antibiotic, typically prescribed for serious infections, such as life-threatening methicillin-resistant *Staphylococcus aureus* (MRSA) skin infections. This drug is also effective against certain protozoa, mycoplasmas, and aerobic and anaerobic Gram-positive bacteria and is mostly utilized in the management of skin infections by preventing protein synthesis or ribosomal translocation [43,44]. By comparing the absorbance values with the standard curve of clindamycin, the dextran hydrogel showed about 70% drug loading capacity after soaking the gel for 2 h in clindamycin solution. These results are in agreement with the previously reported literature where polydopamine-incorporated dextran hydrogels were used as a drug carrier for wound healing and exhibited 71% drug-loading efficiency [31]. It has been documented previously that the effectiveness of drug loading and trapping is mostly influenced by the ionic interaction of the ionic groups of the drug and the hydrogels [37]. In addition to these factors, temperature and the equilibrium swelling degree of the hydrogel play major roles in drug loading [33]. The high drug loading capacity of our dextran hydrogel may be attributed due to the higher swelling ratio and lower crosslink density of the synthesized hydrogel.

#### 2.9.2. In Vitro Drug Release Analysis

**Figure 11:**
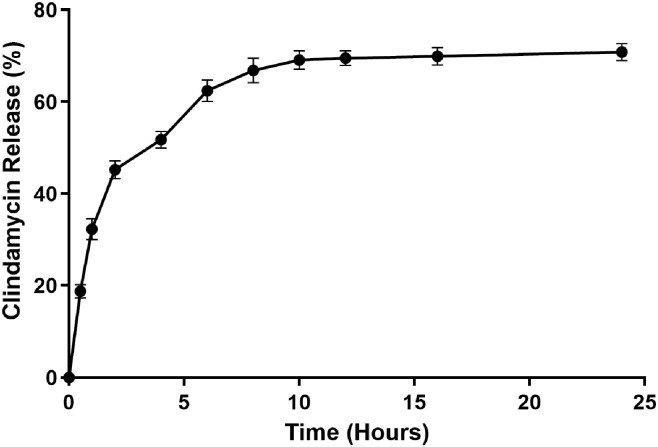
Analysis of in vitro drug release studies of clindamycin from DexH-Clin hydrogel at 450 nm in PBS. All experiments were performed in triplicate (*n* = 3). The data is presented as mean ± SEM.

In vitro drug release studies of clindamycin-loaded dextran hydrogel (DexH-Clin) involve placing the drug-loaded dextran hydrogel in a controlled release environment, typically a buffer solution that mimics physiological conditions. In this study, in vitro drug release studies of DexH-Clin were carried out in PBS buffer, pH 7.4, at 37 °C. The drug release was monitored at various time points by measuring the absorption peaks at 450 nm using UV–visible spectroscopy. The drug release curve revealed that 30% of drug release was achieved within 60 min, followed by a steady slower release pattern which resulted in the release of half of the loaded drug in 4 h. The maximum release (65 ± 2) % of clindamycin into the surrounding buffer was observed after 10 h, after which no further significant drug release was observed (Figure 11). These results highlighted that the dextran hydrogel possesses a rapid drug release capacity of approximately 65% of the loaded drug within 10 h, while 35% of the drug was still retained in the gel matrix.

The phenomenon of drug release may be explained by the different interactions (hydrogen bonds and electrostatic interactions) between polymeric networks of the dextran hydrogel and hydrophilic clindamycin drug molecules. As previously reported, these are crucial for drug release, in addition to affecting the swelling behavior of polymeric hydrogels, and ensure the sustainable release of drugs [31,37].

### 2.10. FTIR Analysis

**Figure 12:**
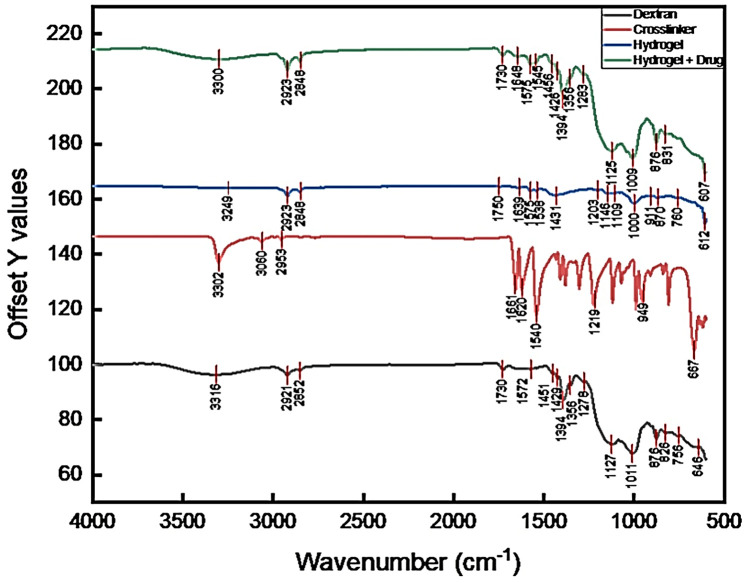
FTIR analysis of dextran hydrogel loaded with clindamycin. Gray = dextran; red = crosslinker; blue = hydrogel; green = hydrogel + drug. Traces are baseline-corrected, normalized and vertically offset for clarity (offset axis labeled). Red vertical ticks mark the peak positions used for assignment of peaks. Key bands: ~3300 cm^−1^ (O–H stretch), 2920/2850 cm^−1^ (aliphatic C–H), 1730 cm^−1^ (C=O where present), 1260–1120 cm^−1^ (C–O–C/saccharide backbone), and 1120–1000 cm^−1^ (polysaccharide fingerprint).

The FTIR spectra of the dextran, crosslinker, synthesized hydrogel, and drug-loaded hydrogel (Figure 12) confirmed the successful formation of the polymeric network and the incorporation of the drug within the matrix. The native dextran spectrum exhibited a broad band around 3316 cm^−1^, attributed to the stretching vibration of hydroxyl (–OH) groups. The O–H stretch at 3316 cm^−1^ is lower than several literature values (~3400–3434 cm^−1^); however, the lower OH frequency could be attributed to stronger hydrogen bonding, typically from more bound water, or indicate extensive hydrogen bonding within the polysaccharide backbone [45]. The absorption peaks at 2921 cm^−1^ and 2852 cm^−1^ corresponded to asymmetric and symmetric C–H stretching vibrations of CH_2_ groups, respectively. The bands at 1730 cm^−1^ and 1640 cm^−1^ were assigned to C=O stretching vibrations and absorbed water bending, while those at 1451–1394 cm^−1^ originated from C–H bending and C–O deformation modes. The characteristic polysaccharide fingerprint bands observed at 1127, 1011, and 876 cm^−1^ were due to C–O–C glycosidic stretching, confirming α-(1→6) linkages typical of dextran. The values of main characteristic bands in this study are lower than the reported values of 1154, 1103, 1020 cm^−1^ and 906 cm^−1^ [46,47,48]; however, this modest shift could be attributed to different factors like the hydration state of the sample; microstructural differences (branching), which varies according to the source of the polymer; measurement mode; and routine instrumental factors. Nonetheless, the FTIR spectrum confirms the dextran polymer has a strong polysaccharide fingerprint.

The crosslinker spectrum exhibited distinctive absorption peaks at 3302 cm^−1^ (–OH/N–H stretch) and 2953–2850 cm^−1^ (C–H stretching), and a strong carbonyl band at 1750 cm^−1^, indicating the presence of ester groups, along with additional bands at 1661–1540 cm^−1^ due to C=O and N–H bending vibrations [49]. Upon hydrogel formation, noticeable shifts and merging of peaks were observed. The O–H stretching band broadened and shifted from 3316 cm^−1^ in dextran to 3249 cm^−1^ in the hydrogel, suggesting enhanced hydrogen bonding between the hydroxyl groups of dextran and functional groups of the crosslinker. A new or intensified absorption at 1759–1639 cm^−1^ in the hydrogel confirmed the formation of ester or amide linkages through the crosslinking reaction. Meanwhile, characteristic C–O–C stretching bands appeared around 1203–1109 cm^−1^, slightly shifted compared to pure dextran, validating the formation of covalent bonds and network crosslinks.

In the drug-loaded hydrogel, additional spectral changes were evident. The broad O–H/N–H stretching band at 3300 cm^−1^ became more intense, and new peaks appeared at 1576–1548 cm^−1^, possibly corresponding to the aromatic or amide functionalities of the encapsulated drug. The C=O band shifted slightly to 1730 cm^−1^, suggesting hydrogen bonding or electrostatic interaction between the drug and the hydrogel network. The persistence of the characteristic dextran peaks at 1011, 906, and 833 cm^−1^ confirmed that the polysaccharide backbone remained intact after drug loading. Overall, the slight shifts and variations in band intensities across spectra demonstrate successful crosslinking, hydrogel network formation, and drug incorporation through hydrogen bonding and weak physical interactions, without altering the fundamental dextran structure. Some major FTIR peaks analyzed and their potential sources in the molecule are listed in Table 1.

### 2.11. Antibacterial Assays of Clindamycin-Loaded Dextran Hydrogel (DexH-Clin)

**Figure 13:**
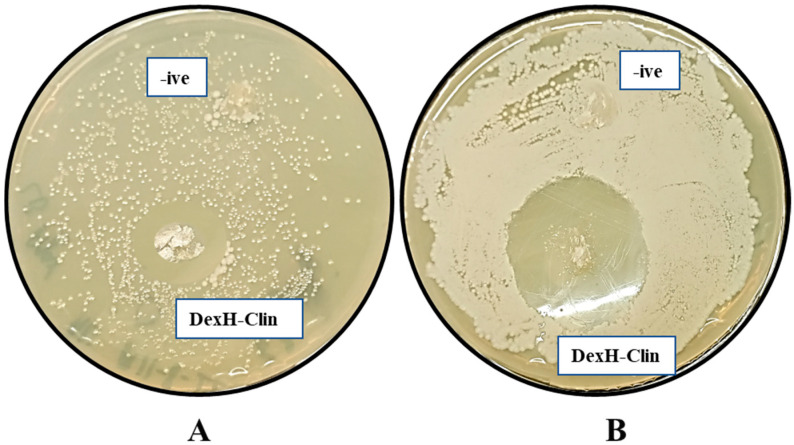
Analysis of antibacterial potential of clindamycin-loaded dextran hydrogels (DexH-Clin) against pathogenic strains using the agar plate method. (**A**): Against *S. gallinarium*; (**B**): against *B. subtilis*.

The antibacterial activity of clindamycin-loaded dextran hydrogels (DexH-Clin) was evaluated against pathogenic *B. subtilis* and *S. gallinarium* strains. Clear zones of inhibition on LB agar plates were observed and measured using a scale. The results demonstrated that DexH-Clin hydrogels exhibited effective drug release and significant inhibitory activity against the tested pathogens. Specifically, the zone of inhibition measured 26 ± 1 mm against *S. gallinarium*, while a substantially larger zone of 37 ± 1 mm was observed for *B. subtilis*, indicating higher susceptibility. In contrast, the pure dextran hydrogel without clindamycin, used as a negative control, showed no inhibitory effect on the growth of either pathogenic strain (Figure 13).

The antibiotic clindamycin has a high level of activity against Gram-positive aerobic bacteria as well as a wide variety of anaerobic bacteria, including beta-lactamase-producing pathogens [50]. It is reported to effectively treat severe skin and soft tissue infections, particularly wound infections that are caused by *Staphylococcus* sp. [51]. In the present study, *B. subtilis* and *S. gallinarum* were selected as representative test organisms to illustrate the performance of clindamycin-loaded dextran hydrogel. *B. subtilis*, a Gram-positive model bacterium, is commonly used in antimicrobial screening and functional meta genomic studies [52]. Clindamycin is primarily active against Gram-positive bacteria and works by inhibiting protein synthesis at the 50S ribosomal subunit. Therefore, testing against *B. subtilis* provides a reliable indicator of whether the drug retains bioactivity after encapsulation and release from the polysaccharide-based hydrogel matrix. *S. gallinarum* mostly causes infections in poultry and frequently forms biofilms that are challenging to cure [53]. Inclusion of such a strain may serve to explore the broader applicability of the hydrogel system beyond human medicine, for example, in veterinary and agricultural contexts (poultry wound care, farm-animal medicine), where cost-effective, stable antibiotic-release dressings are often needed. The formation of a clear zone of inhibition served to illustrate the effectiveness and drug release from the clindamycin-loaded dextran hydrogel against these two pathogens.

Previously, pectin–chitosan hydrogels loaded with ciprofloxacin have exhibited effective antibacterial and wound-healing activity, highlighting the capability of natural-polymer matrices to deliver antibiotics in a moist, biocompatible environment [54]. In addition, non-antibiotic systems such as silver-nanoparticle-based hybrid hydrogels (e.g., lignin–Ag/PVA/alginate) provide broad-spectrum antibacterial activity, illustrating alternative strategies when antibiotic use is limited [55].

In this context, our clindamycin-loaded dextran hydrogel stands out because it combines the biocompatibility and polysaccharide-based matrix advantages of natural-polymer hydrogels with targeted antibiotic delivery. Unlike metal-nanoparticle hydrogels—which may raise cytotoxicity concerns—our system relies on a clinically used antibiotic, potentially reducing toxicity risks while preserving efficacy. Compared with other antibiotic-loaded polymer hydrogels, our dextran-based hydrogel offers a simpler matrix and potentially easier production, while still achieving antimicrobial activity. The pathogen inhibitory potential lies in the range of higher inhibitory activity (>15 mm) against tested pathogenic strains [56], pointing to the high potential of dextran hydrogels for the release of the encapsulated drug. The inhibition of pathogens may be due to the bacteriostatic effects of clindamycin [55]. To the best of our knowledge, it is the first study in which clindamycin was loaded into dextran hydrogel as a pharmaceutically active substance. Thus, our results reinforce and expand upon the trend, documented in the recent literature, that natural-polymer hydrogels can serve as effective, biocompatible antibiotic carriers for wound-healing applications.

## 3. Conclusions

The findings of this study depict that the microbial dextran-based hydrogel has remarkable potential as a drug delivery system, particularly when loaded with clindamycin. The high swelling properties of dextran hydrogel are indicative of its ability to absorb and retain a substantial amount of liquid, making it a promising platform for controlled drug release applications. The ability to efficiently encapsulate and release clindamycin is essential in optimizing therapeutic outcomes while minimizing potential side effects. The clindamycin–dextran hydrogel holds promise as a novel therapeutic option for the treatment of localized bacterial infections and as a platform for the development of other drug-loaded hydrogel systems for applications ranging from wound healing (particularly burn wounds) and tissue engineering to targeted drug delivery systems. These in vitro results are encouraging; however, cytotoxicity and in vivo evaluations are still needed to fully establish the biocompatibility, safety, and clinical relevance of the proposed hydrogel system. Additionally, exploring compatibility with other therapeutic agents will further validate the translational potential of this system. Overall, the results presented in this study provide a strong foundational framework for the development of innovative, cost-effective hydrogel-based drug delivery systems with improved efficacy and controlled release characteristics.

## 4. Materials and Methods

### 4.1. Microorganism and Dextran Production for This Study

Due to its wonderful gelling properties, microbially produced dextran was employed to execute this experiment. Dextran was produced by a high-yielding *Wiesella confusa* B4 strain. This strain has been previously isolated from the gastrointestinal tract of butterflies, identified by 16S rRNA gene sequence analysis and submitted to NCBI under the GenBank Accession No. ON909759.1 (data to be published separately). Dextran was composed of glucose monomers exhibiting 97% α (1→6) linkages in the main chain and 3% α (1→3) branch linkages.

For the production of dextran, de Man Rogosa Sharpe (MRS) medium was prepared as described previously [22,23,57]. The isolate B4 was initially grown in 5 mL MRS medium supplemented with 20% sucrose at 25 °C in a still incubator for 16 h. A 1.0 mL aliquot of this pre-culture was inoculated into 100 mL of MRS–sucrose medium and incubation was performed for 96 h in similar conditions to obtain the highest EPS yield after the maximum utilization of the sucrose substrate.

The *W. confusa* B4 culture was centrifuged at 6000× *g* for 20 min as part of the downstream processing to separate the bacterial cells. The cell pellet was discarded, the supernatant was collected, and the EPS-associated proteins were separated with trichloroacetic acid (TCA) treatment according to the protocol outlined by Abid et al. (2019) [58]. The dextran polymer was precipitated by adding one volume of ice-chilled absolute ethanol, and the precipitated polymer was separated through centrifugation at 6000× *g* for 15 min. The supernatant was discarded and the pellet of precipitated polymer was redissolved in distilled water. Ethanol precipitation was repeated 3 times to ensure the removal of residual sucrose and glucose impurities from EPS. The purity of the dextran was confirmed by thin layer chromatography (TLC) by following the previously described protocol [22,59]. Finally, the EPS pellet was redissolved in distilled water and subjected to freeze drying in order to obtain dextran in fine powdered form [60].

### 4.2. Formulation of Dextran Hydrogel (DexH)

In order to synthesize the dextran hydrogel, *N*,*N* methylene bis-acrylamide (MBA) was used as a crosslinking agent. For the formulation of hydrogel, a previously reported methodology [30] was followed with some modifications. Briefly, 0.4 g dextran was added into 10 mL of 2.8 mol/L aqueous NaOH solution and subjected to continuous stirring for 30 min using a magnetic stirrer to ensure the complete dissolution of dextran. During continuous stirring at room temperature, 0.15 g MBA was added and stirred for further 2 h at a slow speed. The solution was turned into hydrogel in ten minutes after the stirring was halted.

The crosslinking reaction further proceeded, and the formulated dextran hydrogel (DexH) was additionally incubated for 6 h at 25 °C to ensure complete network formation. Following gelation, the gel was immediately immersed in deionized water and neutralized through repeated water exchanges until the wash solution reached neutral pH. This step was repeated 5–6 times, each time with an equal volume of fresh distilled water to ensure complete removal of residual alkali and unreacted chemicals. The physical appearance of the DexH was observed, and the gel was vacuum-dried to obtain it in water-free form.

### 4.3. Effect of Crosslinker Concentration on the Synthesis of Dextran Hydrogel

In order to determine the minimal crosslinker concentration required to create the hydrogel, the crosslinking reaction between MBA and dextran was performed at various MBA concentrations. Four dextran hydrogels with different crosslinker densities were prepared using Dex/MBA *w*/*w* ratios of 1:0.20 (designated as DexH1), 1:0.35 (designated as DexH2), 1: 0.5 (designated as DexH3) and 1: 0.75 (designated as DexH4). Subsequently, the physical characteristics and stiffness of the hydrogels were investigated.

### 4.4. Determination of Water Absorbency and Swelling Kinetics of Dextran Hydrogels

The gravimetric method was used to determine the swelling degree of hydrogels. For this purpose, 0.5 g of dried hydrogel was weighed and immersed in deionized water. The swollen gel was taken out of the water at various time intervals and weighed after blotting with filter paper. The following equations were used to compute the equilibrium swelling ratio (Q) [30] and % swelling (S%) of the synthesized dextran hydrogels [36].

Q = (W_e_ − W_d_)/W_d_

S% = [(W_e_ − W_d_)/W_d_] × 100

where W_d_ is the dry weight of the hydrogel, whereas W_e_ is the weight of swollen hydrogel at different time points.

### 4.5. Effect of pH and Temperature on the Swelling of the Dextran Hydrogel

The effect of pH on the swelling behavior of dextran hydrogel was studied using buffers of different pH levels. Three different buffers, i.e., acetate buffer at pH 5.5, Phosphate-Buffered Saline (PBS) at pH 7.4 and the Tris/HCl buffer at pH 9.0, were used for this purpose. To access the swelling of dextran hydrogel at different temperatures, dried pieces of hydrogel were swollen in dIH_2_O at 25 °C, 30 °C, 37 °C. 45 °C, and 52 °C separately. The equilibrium swelling ratio (Q) and % swelling were calculated using the above-mentioned equations. All experiments were performed in triplicate.

### 4.6. Analysis of Water Retention Capacity of Dextran Hydrogel

In order to determine its water retention capacity, the fully swollen dextran hydrogel was dehydrated at 37 °C. The weight of the hydrogel was measured at different time intervals (t). The following formula was used to determine the water retention capacity of the hydrogel:

Water retention = (W_t_ − W_d_)/W_e_ × 100

where W_d_ (mg) denotes the weight of the dry hydrogel, W_e_ (mg) denotes the weight of the fully swollen hydrogel at equilibrium state, and W_t_ (mg) is the weight of the hydrogel at different time intervals (t).

### 4.7. Estimation of Stability of Hydrogel

In order to determine the stability of the hydrogel, fully swollen hydrogels were immersed in 20 mL PBS and incubated at 37 °C. Samples were taken at 24 h intervals and blotted with filter paper for the weighing calculation. At each sampling time point, the entire medium was removed and replaced with fresh PBS to maintain sink conditions. No mechanical agitation was applied (static incubation). Their degradation rates were determined by calculating the difference between the initial and final weights of the hydrogel. Samples were tested in triplicate (*n* = 3) and results are reported as mean ± SD.

### 4.8. Morphological Characterization of Dextran Hydrogels Using Scanning Electron Microscopy

The morphology of dextran hydrogel was determined using a scanning electron microscope (SEM; Nova, NanoSEM 450 (JEOL, JSM-1010, Tokyo, Japan) at different magnifications. For sample preparation, the freeze-dried hydrogels were coated with a thin film (5–10 nm) of gold using a sputter coater (Desk V HP, Denton Vacuum, Moorestown, NJ USA). The samples were fixed on a copper stud using double-ended carbon tape for electron microscopy analysis.

### 4.9. Drug Delivery Studies

Trials were performed to encapsulate a drug (clindamycin–HCl (antibiotic)) inside the dextran hydrogel to access its potential as a drug delivery vehicle. The absorption spectrum of clindamycin–HCl was obtained in dIH_2_O due to its water-soluble nature. A standard curve was constructed using different concentrations to analyze the encapsulation efficiency and in vitro drug release profile.

#### 4.9.1. Drug Loading Experiment

The drug loading solution of clindamycin HCl was prepared in deionized water with a concentration of 2 mg/mL. After 2–3 h, the hydrogels were taken out of the solutions and blotted with filter paper to remove extra solution for accurate measurement of the hydrogel weight. A UV-vis spectrophotometer (Camspec M350, Cambridge, UK) was used to measure the absorbance of clindamycin at 450 nm in order to assess the drug loading capacity. The readings were compared with the standard curve of the drug. The drug encapsulation efficiency (EE) at the equilibrium state was measured using the following equation as reported in the literature [31].

EE = (C_0_ − C_e_) V/m

where C_0_, and C_e_ (mg/L) are the concentrations of antibiotic at initial time t and equilibrium; m (mg) represents the weight of the hydrogel; and V (mL) is the volume of the antibiotic solution.

The percent encapsulation efficiency (EE%) is calculated using the following formula:

EE(%) = (amount of drug encapsulated/initial drug amount) × 100.

#### 4.9.2. In Vitro Drug Release Studies

The clindamycin–HCl-loaded hydrogel was further tested to determine its in vitro drug release profile. For this purpose, drug-loaded hydrogel was immersed in 5 mL of PBS buffer solution and incubated at 37 °C. Then 1 mL of drug release solution was taken out after each hour and assayed spectroscopically to determine the amount of released drug using a UV–visible spectrophotometer at 450 nm. The whole experiment was performed in triplicate. The amount of released drug was estimated by comparing the concentration from the standard curve of the drug recorded in PBS.

### 4.10. FTIR Analysis of Drug-Loaded Hydrogel

Potassium bromide (KBr) was used as the medium for sample preparation for FTIR analysis of pure dextran, crosslinker, hydrogel, and then drug-loaded hydrogel due to its high transparency in the infrared region and lack of interference with sample spectra. The dry KBr 0.2 g was mixed with approximately 2 mg of finely ground solid samples and the mixture was compressed into a pellet using a hydraulic press at 4 atm for 2–5 min. This process ensures that the resulting absorption spectrum is attributable solely to the sample, producing a uniform, optically clear disk suitable for analysis. The pellet was carefully removed and placed in the FTIR instrument (Thermo Nicolet Nexus 470 FTIR, Austin, TX, USA) for spectral analysis. Spectra were acquired from 4000 to 600 cm^−1^, with 64 co-added scans, maintaining identical parameters for all samples. Peaks were assigned focusing on the polysaccharide O–H stretch (~3300–3400 cm^−1^), C–H stretches (2925–2850 cm^−1^), carbonyl/H–O–H region (~1750–1600 cm^−1^), CH bending (1450–1410 cm^−1^), and the dextran fingerprint cluster (~1200–1000 cm^−1^) including the α-glycosidic band near 906–908 cm^−1^; band shifts in the hydrogel and drug-loaded hydrogel were interpreted in terms of crosslink formation and hydrogen-bonding interactions. All measurements were performed in triplicate at room temperature (22–25 °C).

### 4.11. Antibacterial Assay

The antibacterial activity of the clindamycin-loaded hydrogels (DexH-Clin) was tested against *B. subtilis* and *S. gallinarium* strains. The agar plate method was used for this purpose, where LB agar plates were prepared (1 g/L tryptone, 1 g/L NaCl, 0.5 g/L yeast extract and 2.5 g/L agar) in deionized water. The medium was sterilized at 121 °C for 20 min, poured into the Petri plates and allowed to solidify under sterilized conditions. After that, 50 µL of overnight grown culture of each tested strain was dispersed onto separate agar plates under aseptic conditions. Dextran hydrogels loaded with clindamycin were placed on the surface of a solid agar medium, whereas water-swollen hydrogels were used as a negative control. The plates were incubated aerobically for 16 h at 37 °C, after which zones of inhibitions were measured to assess the antibacterial potential of drug-loaded hydrogels.

## Figures and Tables

**Table 1:** Major FTIR bands observed and assignments.

Wavenumber (cm^−1^)	Assigned Vibration	Rationale/Typical Source
~3300	Broad O–H stretching (hydrogen-bonded hydroxyls; water/–OH of polysaccharide)	Typical for polysaccharides and hydrogels (O–H stretch).
2920–2850	C–H asymmetric/symmetric stretching (aliphatic CH_2_/CH_3_)	Common in sugars and crosslinkers.
~1730	C=O stretching (ester/carbonyl)	Present if crosslinker introduces ester groups or solvent residues.
1650–1600	H–O–H bending/amide I (if proteins present)/C=C conjugated modes	Interpret in context of sample composition.
1575–1500	Asymmetric COO^−^ or aromatic ring modes (depends on crosslinker)	Check crosslinker chemistry.
1425–1375	CH_2_ bending/CH_3_ deformation	Typical skeletal vibrations.
1260–1150	C–O–C and C–O stretching (glycosidic linkages)	Characteristic of polysaccharide backbone and ethers.
1120–1000	Strong C–O stretching/ring vibrations (saccharide fingerprint)	Key carbohydrate region; used to confirm polysaccharide skeleton.
900–700	Anomeric C–H or glycosidic-related bands; ring deformation	Useful to indicate glycosidic linkages or substitution pattern.

## Data Availability

The datasets generated during and/or analyzed during the current study are available from the corresponding authors on reasonable request.

## Supplementary Materials

The following supporting information can be downloaded at: https://www.mdpi.com/article/10.3390/gels12010082/s1, Figure S1. Chemical Structure of Dextran. Figure S2. Calibration curves of clindamycin-HCl in (a) deionized water (b) PBS.
