# Supplementary material for: Development and Characterization of Clindamycin-Loaded Dextran Hydrogel for Controlled Drug Release and Pathogen Inhibition
Source: Gels. 2026 Jan 17;12(1):82. doi: 10.3390/gels12010082 (PMC12841258; doi:10.3390/gels12010082)
Supplement: Supplementary file 1 [file gels-12-00082-s001.zip › gels-4012750-supplementary.pdf]

Supplementary file

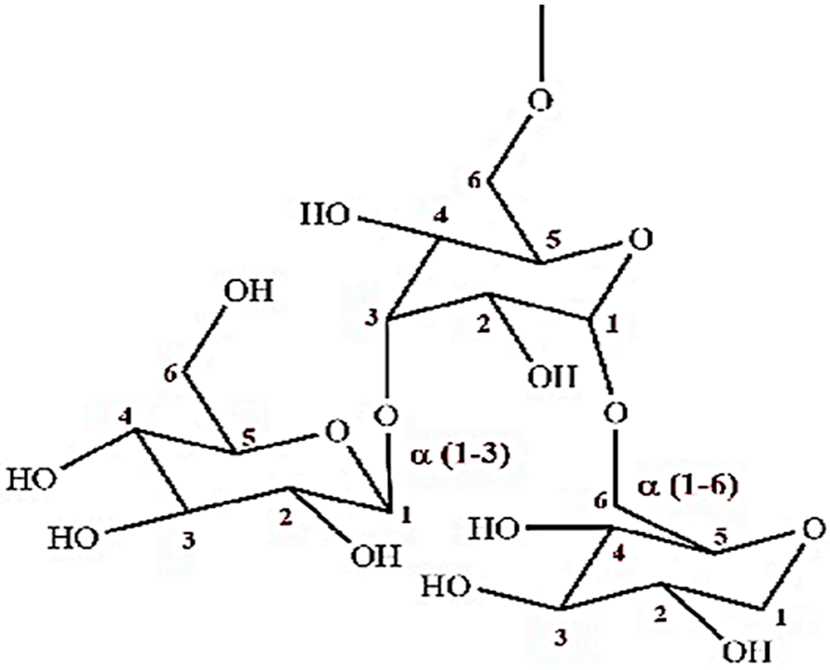

Figure S1. Chemical Structure of Dextran.

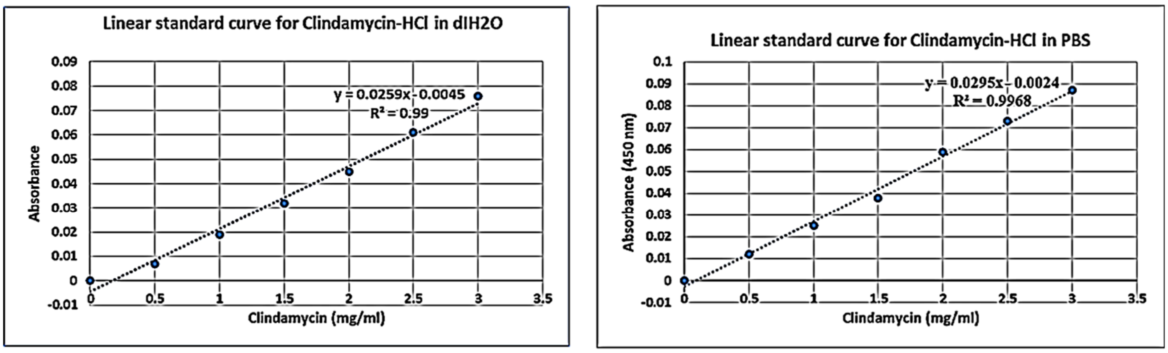

A

B

Figure S2. Calibration curves of clindamycin-HCl in (a) deionized water (b) PBS
